# Update on medical treatment for Cushing’s disease

**DOI:** 10.1186/s40842-016-0033-9

**Published:** 2016-09-13

**Authors:** Daniel Cuevas-Ramos, Dawn Shao Ting Lim, Maria Fleseriu

**Affiliations:** 1 Department of Endocrinology and Metabolism, Neuroendocrinology Clinic, Instituto Nacional de Ciencias Médicas y Nutrición Salvador Zubirán, Vasco de Quiroga 15, Sección XVI, Tlalpan, Mexico City, 14030 Mexico; 2 Departments of Medicine (Endocrinology) and Neurological Surgery, and Northwest Pituitary Center, Oregon Health & Science University, 3303 SW Bond Ave, Mail Code CH8N, Portland, OR 97239 USA

**Keywords:** Hypercortisolemia, Cushing’s disease, Pasireotide, Mifepristone, Ketoconazole, Osilodrostat, Levoketoconazole

## Abstract

Cushing’s disease (CD) is the most common cause of endogenous Cushing’s syndrome (CS). The goal of treatment is to rapidly control cortisol excess and achieve long-term remission, to reverse the clinical features and reduce long-term complications associated with increased mortality.

While pituitary surgery remains first line therapy, pituitary radiotherapy and bilateral adrenalectomy have traditionally been seen as second-line therapies for persistent hypercortisolism. Medical therapy is now recognized to play a key role in the control of cortisol excess. In this review, all currently available medical therapies are summarized, and novel medical therapies in phase 3 clinical trials, such as osilodrostat and levoketoconazole are discussed, with an emphasis on indications, efficacy and safety. Emerging data suggests increased efficacy and better tolerability with these novel therapies and combination treatment strategies, and potentially increases the therapeutic options for treatment of CD. New insights into the pathophysiology of CD are highlighted, along with potential therapeutic applications. Future treatments on the horizon such as R-roscovitine, retinoic acid, epidermal growth factor receptor inhibitors and somatostatin-dopamine chimeric compounds are also described, with a focus on potential clinical utility.

## Background

In 1932, Harvey Cushing described a syndrome characterized by serious manifestations consequent to systemic effects of chronic exposure to cortisol *“…which has been found at autopsy in 6 out of 8 to be associated with a pituitary adenoma…”* [1]. Cushing’s syndrome (CS) results from chronic, excessive exposure to glucocorticoids, the source of which may be either exogenous or endogenous. The most common cause (70 %) of endogenous cortisol production is Cushing’s disease (CD) due to overproduction of adrenocorticotropic hormone (ACTH) by a pituitary corticotroph adenoma [2, 3]. ACTH, in turn, stimulates melanocortin type 2 receptor (MC2R) at the adrenal cortex and increases cortisol synthesis [4, 5]. CD is more frequently observed in women, and in about 90 % of cases, is due to a pituitary microadenoma or corticotroph hyperplasia. Once ACTH-dependent glucocorticoid excess is confirmed, further tests are used to clarify the source of ACTH secretion [2, 6, 7]. Magnetic resonance imaging (MRI) may confirm the presence of a pituitary adenoma, however, in up to 40 % of cases, an adenoma remains undetectable [8, 9]. When a lesion is not visible or appears smaller than 6 mm on imaging, bilateral inferior petrosal sinus sampling is recommended to clearly distinguish between CD and ectopic ACTH production [6].

The goals of treating CD are to eliminate the source of ACTH excess, achieve biochemical eucortisolism and long-term remission, to reverse clinical features, reduce long-term complications and mortality, and improve quality of life [10]. Surgical resection of the identified pituitary adenoma remains first line treatment in CD patients, however, remission rates are reported to be 65–90 % for those with microadenomas and less than 65 % for those with macroadenomas [11, 12]. While repeat pituitary surgery, pituitary radiotherapy or bilateral adrenalectomy have traditionally been used as adjuvant therapies in persistent hypercortisolism [3, 11, 13], medical treatment now plays an increasingly important role in CD management [7, 10, 14]. Novel therapeutic medications and strategies have recently emerged. Such medications are summarized in this review, with emphasis placed on indications, efficacy and safety. New medical treatments on the horizon for CD are described, with a focus on treatments with potential clinical utility in the near future.

### Indications for medical therapy

Over the last 10 years, the armamentarium of drugs available for CD treatment has expanded significantly. Many drugs have been shown to decrease cortisol levels and improve the clinical syndrome, and a reduction in tumor volume has been observed with some [15]. Medical therapy should be considered in the following circumstances: 1) as adjuvant therapy for persistent hypercortisolism after surgery [10, 16]; 2) as a pre-operative treatment in severe cases, [17]; 3) treatment of acute and life-threatening hypercortisolism complications (i.e. sepsis, uncontrolled hypertension, severe hyperglycemia, heart failure, intractable hypokalemia and psychosis [18, 19]; 4) whilst awaiting the full treatment effects of radiotherapy [20]; and 5) as first-line treatment in patients with surgical contraindications, those who decline surgery, in whom no definite lesion is seen, or when tumor is in an unfavorable location [14].

## Medical treatments currently used in Cushing’s disease

Medications currently used in the treatment of CD are classified according to their mechanism of action as adrenal steroidogenesis inhibitors, pituitary-directed drugs and glucocorticoid receptor antagonists [21] (Table 1).

**Table 1:** Medical therapy for Cushing’s disease

Classification	Drug name	Dose	Possible adverse events; Close monitoring is necessary for all drugs
Steroidogenesis inhibitors	Ketoconazole	Oral 200–1200 mg/day 2–3 times a day	Hepatitis, gastrointestinal disturbances, gynecomastia, skin rash, adrenal insufficiency.
	Fluconazole	Oral 100–200 mg/day 2 times a day	AEs similar to ketoconazole, not well studied
	Metyrapone	Oral 0.5–6 g/day 4 times a day	Hirsutism, acne, hypertension, hypokalemia, edema, gastritis, nausea, adrenal insufficiency.
	Etomidate	IV bolus of 0.03 mg/kg followed by 0.02–0.08 mg/kg/h	Somnolence, myoclonus, nausea, vomiting, dystonic reactions, adrenal insufficiency.
	Mitotane	Oral 2–5 g/day 3 times a day	Gastrointestinal disturbances, hepatitis, neurologic manifestations, gynecomastia, neutropenia, lipid disorders, adrenal insufficiency; teratogenic
	*Osilodrostat*	*Oral 10–60 mg/day; phase III clinical trial: NCT 02180217*	*Nausea, asthenia, diarrhea, adrenal insufficiency. Testosterone increase in women.*
	*Levoketoconazole*	*Oral 400 mg /day; phase III clinical trial: NCT 01838551*	*N/A.*
Dopamine D2R agonists	Cabergoline	Oral 0.5–7 mg/week	Headache, nausea, dizziness, nasal congestion, hypotension, depression
SRLs	Pasireotide^a^	SC 300–1800 µg/day 2 times a day	Hyperglycemia, diabetes, diarrhea, nausea, abdominal pain, cholelithiasis, QT prolongation
	*Pasireotide LAR*	*IM 30–60 mg/month; phase III clinical trial*	
GR antagonist	Mifepristone^a^	Oral 300–1200 mg/day Once daily	Nausea, fatigue, headache, hypokalemia, arthralgia, vomiting, peripheral edema, hypertension, dizziness, adrenal insufficiency, PAECs, endometrial thickening, vaginal bleeding; termination of pregnancy.
*CDK2/Cyclin E inhibitor*	*R-Roscovitine*	*Oral Phase II clinical trial; clinicaltrials.gov identifier: NCT02160730*	
*Nuclear receptor ligand*	*Retinoic acid*	*Oral Phase II clinical trial; clinicaltrialsregister.eu number: 2008-006379-65*	

*D2R* dopamine D2 receptor, *SRL* somatostatin receptor ligand, *SC* subcutaneous, *IM* intramuscular, *LAR* long-acting repeatable, PAECs- progesterone-receptor modulator-associated endometrial changes

^a^FDA approved

### Adrenal steroidogenesis inhibitors

Adrenal steroidogenesis inhibitors block cortisol synthesis by inhibiting multiple key enzymes involved in steroidogenesis. Cortisol levels decrease, but no effect is observed on the underlying corticotroph tumor. There are no prospective studies on the efficacy and safety of steroidogenesis inhibitors, and variability in study design and quality makes comparison of efficacy difficult [22, 23]. Currently, apart from metyrapone and ketoconazole, which are approved in the European Union (EU), steroidogenesis inhibitors are used as off-label therapy in most other countries. Cortisol excess may be rapidly controlled but the “escape phenomenon” is common and higher doses are needed in the long-term to achieve disease control. They are therefore most commonly used temporarily as pre-operative treatment in patients with severe complications from CS when rapid control is necessary, and as a bridging therapy after radiotherapy [14, 22, 24]. Two treatment approaches are used: “normalization” of cortisol and the “block and replace” strategy that involves complete blockage of cortisol production and replacement with physiological doses of glucocorticoids.

Because of their effects on hepatic cytochrome p450 expression, drug interactions are commonly seen with adrenal steroidogenesis inhibitors. There is also limited experience with these medications during pregnancy and as Cushing’s disease affects mostly women of reproductive age, caution is advised in women planning pregnancy. Individual drug dosing, frequency and adverse effects are summarized in Table 1.

#### Ketoconazole

Ketoconazole is an imidazole derivative first used as an anti-fungal therapy. It was noted, however, that patients developed adrenal insufficiency at high doses. Ketoconazole inhibits steroidogenesis by blocking cholesterol side-chain cleavage complex, 17,20-lyase, 11beta-hydroxylase and 17 alpha-hydroxylase enzymes [25]. It has been postulated that ketoconazole also has an inhibitory effect on corticotroph ACTH secretion, though this remains debatable [26]. In the largest retrospective study to date (FReSKO), 200 patients received ketoconazole at doses of 200–1200 mg daily for pre-operative, primary and secondary treatment of CD. At last follow-up (mean 20.6 months), about half achieved normal urine free cortisol (UFC) levels and an additional 26 % had a ≥ 50 % decrease, associated with clinical improvement. Escape from treatment however, was demonstrated in 23 % of those with initial response [27]. Of note, like in previous reports, intolerability resulted in treatment cessation in approximately one-fifth of patients. Though generally reversible upon drug withdrawal, up to 5-fold increases in liver enzymes were found in 13.5 % of patients while severe hepatotoxicity (>5-fold elevation) was observed in 2.5 %. It is recommended that liver function be closely monitored during treatment with ketoconazole and dose reduction or discontinuation is advised when liver enzymes are 3-fold elevated [10]. More recently, use of ketoconazole as a combination therapy has been studied (see below).

#### Fluconazole

Fluconazole was found in in vitro studies to inhibit cortisol production in human adrenocortical cells via inhibition of 17-hydroxylase and 11-beta hydroxylase. The effect was shown to be dose-dependent; potency was lower than ketoconazole. Though potentially promising and with less risk for hepatotoxicity, there are few reports of fluconazole use demonstrating clinical efficacy [28, 29].

#### Metyrapone

This steroidogenesis inhibitor primarily blocks 11-beta hydroxylase, as well as the cholesterol side chain cleavage complex, 17-alpha hydroxylase and 18-hydroxylase enzyme activities. Studies demonstrate normalization of cortisol secretion in 45–75 % of patients [17, 30, 31]. In the largest and most recent multicenter retrospective study to date, 195 patients with CS (59 % CD) were treated with metyrapone for a mean duration of 8 months. The majority was being treated pre-operatively for control of severe CS; 35 patients were on combination therapy. In patients on monotherapy, 55 %, 46–76 %, and 43 % of patients achieved biochemical control as assessed by a normal cortisol day curve, morning cortisol and UFC, respectively. Longer-term data (mean 18 months) available for 38 patients showed biochemical eucortisolism in 72 % [32].

Overall, escape has been reported in up to 19 % of patients with an initial response [14]. In addition, ACTH levels frequently increase and may drive androgen and mineralocorticoid overproduction, causing multiple adverse events (Table 1). Mineralocorticoid overproduction occurs due to 11-beta hydroxylase inhibition and accumulation of aldosterone precursors. To avoid mineralocorticoid-related adverse events, metyrapone is therefore often used in the short-term, for pre-operative control and in severe CD in combination with ketoconazole [33], though cases of long-term use have been reported [34].

#### Etomidate

Similar to other adrenal-directed drugs, etomidate acts by blocking multiple enzymes involved in steroidogenesis such as side-chain cleavage complex, 17-hydroxylase, 11 beta-hydroxylase, 17–20 lyase enzymes and aldosterone synthase [35]. After intravenous administration, a rapid and significant suppression of serum cortisol levels is seen within 5 h and maximal suppression occurs at 11 h [36–39]. Etomidate is therefore used to rapidly control severe manifestations of CS such as psychotic crises, severe sepsis or hypertension, in patients who are not immediate surgical candidates and are unable to tolerate oral medications [10, 40, 41]. Use of etomidate, however, should be limited to the intensive care setting, with close monitoring for central nervous system depression. Multiple regimens have been studied to block steroidogenesis whilst trying to avoid its anesthetic effects. Various approaches for complete and partial blockade also exist [40–42]. Guidelines published by the Endocrine Society suggest a loading dose followed by a weight-based continuous infusion titrated to achieve serum cortisol levels between 10 and 20 μg/dL (280–560 nmol/L) [10]. It has, however, also been suggested that treatment targets in the intensive care setting, be higher than in the non-acute setting at 18–30 μg/dL (500–800 nmol/L) [41]. Serum electrolytes and cortisol levels therefore need to be monitored every 4–6 h, to achieve target cortisol levels and to prevent adrenal insufficiency, which may require treatment with intravenous hydrocortisone (“block and replace” regimen) [14].

#### Mitotane

Mitotane (o,p’-diphenylmethane derivative agent) has both adrenostatic and adrenolytic actions, and is for this reason, most commonly used in the treatment of adrenocortical carcinoma. Mitotane blocks the cholesterol side-chain cleavage complex, 11 beta-hydroxylase, 18-hydroxylase and 3 beta-hydroxysteroid-dehydrogenase (HSD3B2) enzymes, reducing cortisol production [43]. Due to its slow onset of action, efficacy is delayed; therapeutic levels are achieved in up to 3 months. Remission rates up to 100 % have been reported, however, concomitant use of pituitary irradiation is likely to have confounded these results [14]. A single-center French retrospective study of 67 patients with CD demonstrated remission in 72 % after treatment for a median duration of 6.7 months. Adverse events were frequent, including gastrointestinal disturbance (50 %), impaired mentation and dizziness (30 %) [44]. Mitotane’s long half-life (up to 160 days) also results in significant circulating drug levels despite drug continuation [43]. Supraphysiological doses of hydrocortisone replacement are often necessary to treat adrenal insufficiency as mitotane increases cortisol-binding globulin (CBG) and activates cytochrome P450 3A4 CYP3A4, resulting in accelerated metabolism of exogenous steroids [45].

### Pituitary-directed drugs

This group of medications acts directly on corticotroph adenomas to reduce ACTH secretion. Two currently available classes of medications that act centrally are dopamine type 2 receptor (D2R) agonists, and somatostatin receptor ligands (SRL). Dose regimens and adverse events are summarized in Table 1.

#### Dopamine D2 receptor agonists

About 80 % of corticotroph pituitary adenomas demonstrate D2R expression [46, 47]. The two available dopamine agonists are bromocriptine and cabergoline; studies have shown variable short- and long-term effectiveness and adverse effects of bromocriptine, thus limiting its use [14].

Small retrospective and open-label prospective studies in patients with persistent CD after surgery demonstrate biochemical response to cabergoline in up to 75 % of patients, though UFC normalization within 3–6 months has only been reported in 25–40 % [48–51]. The “escape phenomenon” is not uncommon, and reported in up to one-third; long-term control of cortisol secretion over 12–24 months was demonstrated in 30–40 % of patients on cabergoline at a median dose of 2–3.5 mg/week [49, 50]. Though it has been suggested that efficacy improves with higher cabergoline doses [49–51], no consistent dose-dependent reduction in cortisol levels was seen in a short-term prospective study of 20 patients on a median dose of 5 mg/week [52]. Tumor shrinkage is also rare, reported in 20 % of cases [50]. As the data regarding cabergoline’s efficacy is at present still limited, its use in the treatment of CD remains off-label [14]. Cabergoline is fairly well tolerated, and thus far, no significant association has been found between its use for the treatment of pituitary tumors and the development of clinically significant cardiac valve disease [53].

#### Somatostatin receptor ligands (SRL)

Somatostatin inhibits ACTH synthesis and secretion. Corticotroph adenomas commonly express somatostatin receptors (SSTR), mainly type 2 (SSTR2) and 5 (SSTR5) [54, 55]. However, hypercortisolism reduces expression of SSTR2, and classical SRLs that act mainly on SSTR2 (octreotide and lanreotide) have not been shown to be effective in patients with CD [56, 57]. Although decreasing cortisol levels may increase SSTR2 expression, this phenomenon occurs at the mRNA level. No increase of SSTR2 protein and receptor externalization has been noted [58]. Further research has led to the development of pasireotide, a multireceptor-targeted SRL with high binding activity to SSTR5, and also to SSTR2, SSTR3 and SSTR1.

#### Pasireotide

In 2012, pasireotide became the first medical treatment to be approved by the European Medicines Association (EMA) and the United States Federal Drug Association (FDA), for the treatment of CD after unsuccessful pituitary surgery or when surgery is contraindicated. In a phase II, open-label, single-arm, multicenter pilot study, 76 % of patients had a reduction in UFC and 17 % achieved complete normalization when subcutaneous (sc) pasireotide was administered at 600 μg twice a day for 15 days. Reductions were also seen in serum cortisol and plasma ACTH levels [59]. This was followed by a phase III, double-blind, randomized clinical trial in which 162 patients were administered 600 or 900 μg of pasireotide twice daily, with dose escalations of 300 μg made after 3 months, depending on UFC response. At 6 months, UFC decreased in the majority; almost half had > 50 % reductions and 20 % achieved normal UFC without dose escalation. Response was rapid, with a median 50 % reduction of UFC in the first 2 months of treatment. Open-label extension through 12 months showed sustained UFC normalization in 13 % of patients in the 600 μg group and 25 % in the 900 μg group. Of note, patients with mild hypercortisolism at baseline had higher UFC normalization rates than patients with severe hypercortisolism [60]. Late-night salivary cortisol (LNSC) was also measured in 93 patients, and normalized in 37 and 19 % of patients who had elevated baseline LNSC levels, at 6 and 12 months, respectively. A moderate correlation (*r* = 0.55) was found between LNSC and UFC measurements [61]. Significant improvement in clinical signs and symptoms was also seen at 12 months of treatment [62]. Additional data from an international expanded-access study also showed UFC normalization in about 40 % of patients who remained on treatment at 48 weeks [63]. In a clinical practice setting study, 48 % of patients with complete UFC normalization at 12 months remained controlled at 24 months, suggesting the possibility of treatment escape in the remainder. Of note, however, is that, despite this, clinical benefits, including blood pressure control and weight reduction were sustained [64]. Data from case reports and small case series suggest that in a limited population, treatment efficacy may extend beyond 5 years [65, 66]. More recently, 60-month follow-up data demonstrate that initial reductions in UFC and clinical improvement were maintained in patients who remained on treatment, suggesting that pasireotide may be effective as a long-term treatment in selected patients with CD [67]. Discontinuation rate was, however, high due to unsatisfactory therapeutic effect (32.7 %), adverse events (22.2 %), consent withdrawal (18.5 %) and administrative problems (9.9 %).

Tumor shrinkage has also been reported; about 9 and 44 % of patients on pasireotide 600 and 900 μg, respectively, showed tumor size reduction at 12 months, suggesting a dose-dependent effect [60]. More recently, significant (>25 %) reductions in tumor volume and even tumor disappearance has also been observed [68]; tumor shrinkage was also reported in corticotroph macroadenomas on *de novo* treatment with pasireotide [69]. Therefore, in addition to its use in patients with persistent or recurrent CD after pituitary surgery, pasireotide may also be useful as first line therapy in macroadenomas to facilitate surgical resection.

With the exception of hyperglycemia incidence and severity, the safety profile of pasireotide is similar to other SRLs [60, 64, 70]. In the pivotal phase III study, hyperglycemia-related adverse events were documented in 73 %, the risk of which was higher in patients with pre-existing diabetes or impaired glucose tolerance. Hyperglycemia was observed soon after the initiation of pasireotide, but glucose and glycated hemoglobin levels stabilized after the initiation or escalation of glucose-lowering therapy. At the end of the study, 48 % of patients who did not have diabetes at baseline had glycated hemoglobin ≥ 6.5 % [60]. Reduction in insulin secretion and the incretin response have been demonstrated in healthy volunteers receiving pasireotide [71]. Metformin and dipeptidyl peptidase 4 (DPP-4) inhibitors are therefore generally recommended as first and second line therapy, respectively for pasireotide-related hyperglycemia [72, 73]. Glucagon-like peptide-1 (GLP-1) agonists and insulin may also be required in cases of more severe glucose intolerance [71, 72, 74].

An intramuscular long-acting-release (LAR) formulation of pasireotide approved for the treatment of acromegaly is now being evaluated for use in CD. Pasireotide LAR was previously reported to be effective in reducing ACTH and tumor volume in a patient with Nelson’s syndrome and aggressive corticotroph tumor growth [75]. In a double-blind, randomized controlled phase III trial of 150 patients with persistent, recurrent or *de novo* CD, UFC normalization was observed in 40 % of patients treated with pasireotide LAR 10 or 30 mg monthly for a duration of 7 months. Median decrease in UFC was 48 % [76]. Safety profile was similar to that of twice-daily sc pasireotide. Hyperglycemia-related adverse events were noted in 68 and 80 % of patients in the 10 and 30 mg groups, respectively, which led to treatment discontinuation in only 5 patients. As pasireotide LAR is administered monthly, improved patient compliance is a potential advantage over sc pasireotide.

#### Other regulators of the hypothalamus-pituitary-adrenal axis

Serotonin antagonists and gamma-amino butyric acid (GABA) agonists are neuromodulatory drugs that have previously been studied in CD, but shown to have limited efficacy, and serious adverse effects [4, 77, 78]. Results from studies analyzing the nuclear receptor ligand, peroxisome proliferator-activated receptor gamma (PPAR-gamma) have also been inconsistent, mainly demonstrating short-term disease control, high rates of treatment escape and minimal clinical improvement [79–81].

### Glucocorticoid receptor antagonists

#### Mifepristone

Mifepristone is a high-affinity, non-selective glucocorticoid receptor (GR) antagonist with ten and four times greater affinity to the GR compared to cortisol and dexamethasone, respectively [82, 83]. GR antagonism results in rapid control of the systemic effects of cortisol excess in patients with CS. ACTH, and subsequently cortisol levels increase due to a loss of negative feedback regulation [84–86]. Such parameters are therefore, not useful for monitoring treatment efficacy or disease activity. Instead, improvement in clinical and metabolic features of CS should be used to evaluate treatment efficacy [14, 87, 88].

Two studies have evaluated the efficacy and safety of mifepristone. In the first retrospective study, 20 patients with CS were treated with mifepristone; four had CD, 3 of whom had rapid improvement in clinical signs and symptoms [89]. Following this was a large prospective multicenter trial, *Study of the Efficacy and Safety of Mifepristone in the Treatment of Endogenous Cushing’s Syndrome (SEISMIC),* which looked at the effects of 24 weeks of mifepristone (300–1200 mg/day) in 50 patients with CS, among whom 43 had CD [84]. In patients with diabetes mellitus or glucose intolerance, significant improvements in glucose profiles were observed in 60 %. Decrease in body weight, waist circumference and fat mass, and increases in insulin sensitivity were observed in 87 % of patients. Post-hoc analysis also demonstrated improvements in global clinical response assessments (based on glucose, lipid and blood pressure control, body composition, clinical appearance, strength, psychiatric/cognitive symptoms and quality of life parameters) in 88 % of patients at 24 weeks [90]. Of 27 patients who entered a long-term extension study (median treatment duration 11.3 months), a dose-dependent ≥2-fold increase in ACTH was observed in 72 %, which returned to baseline after drug discontinuation. Tumor progression was seen in four cases; three had macroadenomas and in one patient, follow-up MRI demonstrated a microadenoma that was not previously detected at diagnosis [91]. Recently, maintenance of weight loss was demonstrated at 2 years follow-up [92]. Based on results of the SEISMIC study, the FDA, in 2012, approved mifepristone for the treatment of patients with endogenous CS who have failed surgery or are not surgical candidates and have concomitant type 2 diabetes mellitus or glucose intolerance [10].

The lack of biochemical parameters for monitoring treatment response to mifepristone is an important limitation especially with regard to assessment for adrenal insufficiency. Adrenal insufficiency was reported in 2 out of 50 patients in the SEISMIC study; another 5 patients were treated with dexamethasone for suspected adrenal insufficiency. Due to mifepristone’s long half-life and high affinity for the GR, supraphysiological doses of dexamethasone (2–8 mg daily in this study) are needed to overcome GR antagonism [22, 93]. High cortisol levels also have the potential to overwhelm renal inactivating enzyme 11 beta-hydroxysteroid dehydrogenase type 2. Subsequent mineralocorticoid receptor activation by excess cortisol results in hypertension, hypokalemia and edema, which often respond well to potassium replacement and mineralocorticoid receptor blockade with spironolactone [10, 84]. Additionally, due to its anti-progestin activity, mifepristone is contraindicated in pregnancy and in women with endometrial hyperplasia or carcinoma, or unexplained vaginal bleeding [14, 89, 94]. A recent case report suggested, however, no endometrial hyperplasia or proliferative changes in a patient with prolonged exposure to ulipristal, another selective progesterone receptor modulator [95]. Caution is also needed with concomitant use of CYP3A4 inhibitors such as ketoconazole, which may increase mifepristone levels, and drugs that increase QT interval, such as pasireotide [94]. Other adverse events are summarized in Table 1.

## Combination drug therapy for Cushing’s disease

As highlighted above, no single drug has demonstrated complete efficacy in the treatment of CD. Adverse events are also not uncommonly encountered in patients on medical therapy (Table 1). A strategy to increase treatment efficacy, whilst reducing doses of individual drugs and thereby minimizing adverse events, is to combine drugs with additive, synergistic, and/or complementary mechanisms of action.

### Combination of adrenal steroidogenesis inhibitors

Of 62 patients with CS (84 % CD) treated pre-operatively with both ketoconazole (200–600 mg/day) and metyrapone (750–1000 mg/day), normalization of UFC was observed in 45 %. Median duration of treatment was 4 months [30]. This combination is also used in the treatment of severe CS when immediate surgery is contraindicated [10, 30, 33]. Additionally, use of 3 adrenal steroidogenesis inhibitors, ketoconazole (400–1200 mg/day), metyrapone (3.0–4.5 g/day) and mitotane (3.0–5.0 g/day), resulted in rapid normalization of UFC and clinical improvement in 11 patients with severe ACTH-dependent CS (4 CD). Ketoconazole and metyrapone were successfully discontinued in 7 patients after 3.5 months; UFC remained normal with mitotane monotherapy. There was, however, a high rate of adverse events with this triple-combination, with acute adrenal insufficiency being reported in 36 % of patients, and liver enzyme elevation in up to 82 % [96].

### Cabergoline and ketoconazole

This treatment approach combines a pituitary-directed drug with an adrenal steroidogenesis inhibitor, therefore targeting both pituitary ACTH and adrenal cortisol synthesis. In a small series, the addition of very low-dose ketoconazole (50–200 mg/day) to cabergoline (3.5 mg/week) resulted in UFC normalization in six patients who had a partial response to cabergoline [97]. Thereafter, in a prospective study of 12 patients with persistent CD after pituitary surgery, the addition of ketoconazole to cabergoline increased rates of UFC normalization from an initial 25 % with cabergoline alone (dose 2–3 mg/week), to 75 % when 200–400 mg/day of ketoconazole was added. Amongst the six patients who benefitted from combination therapy, subsequent cabergoline dose reductions could be made in two patients [51]. Similar findings were demonstrated in a subsequent prospective analysis of 14 patients with persistent, recurrent or de novo CD: combination therapy with cabergoline doses of up to 3 mg/week and ketoconazole up to 600 mg/day resulted in UFC normalization in 79 % of patients. Neither drug was effective as monotherapy, nor did the choice of starting treatment (cabergoline vs. ketoconazole) affect outcomes [98]. Of note, though combination therapy lowered late night salivary cortisol levels, they remained elevated, indicating persistent abnormal diurnal variation. Larger studies are needed to confirm these findings.

### Pasireotide, cabergoline and ketoconazole

The combination of pasireotide, cabergoline and ketoconazole was studied in a prospective open-label study of 17 patients with CD. Patients were placed on sc pasireotide 100–250 μg 3 times/day at the start of the study. If no disease control was achieved at 28 days, cabergoline was added (0.5–1.5 mg every other day), and if UFC remained elevated at 60 days, ketoconazole 200 mg 3 times/day was added. This step wise addition of first cabergoline, and then ketoconazole to pasireotide, increased UFC normalization rates from 29 to 53 to 88 %, respectively at 80 days, with associated decreases in body weight, waist circumference and blood pressure [99]. Quality of life did not change significantly in the short term, but improved after 1 year of remission in three patients who continued medical therapy [100]. A large prospective, phase II open-label pasireotide-cabergoline study is currently under way [101].

## Novel medical treatments for Cushing’s disease in clinical trials

### Osilodrostat (LCI699)

**Fig. 1:**
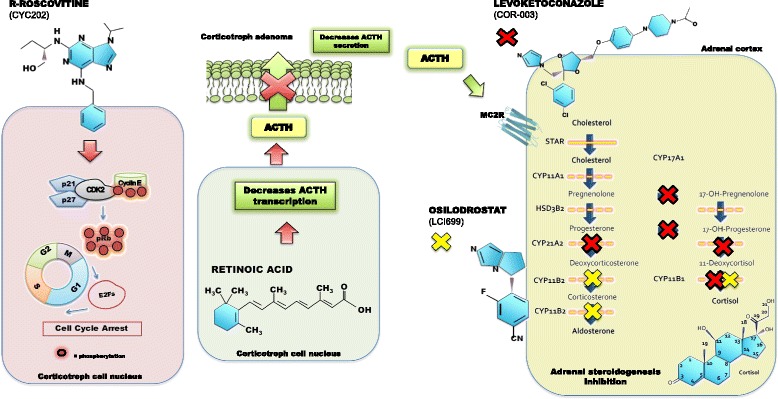
Mechanism of action of novel medical treatments for Cushing’s disease. From left to right: R-roscovitine induces cell cycle arrest, tumor shrinkage and reduction of ACTH synthesis and secretion. Exact mechanism of action is currently under study. Retinoic acid inhibits POMC expression, decreasing ACTH synthesis and release. An additional novel treatment under evaluation is the selective, peptide melanocortin-2 receptor (MC2R) antagonist that blocks ACTH action at the adrenal cortex. Osilodrostat is a steroidogenesis inhibitor that blocks the action of CYP11B1 and CYP11B2 (*yellow* X) with greater affinity than metyrapone. Levoketoconazole is a steroidogenesis inhibitor with greater potency than ketoconazole in blocking CYP11B1, CYP21 and CYP17 during cortisol synthesis (*red* X). Osilodrostat and levoketoconazole are currently being studied in phase III clinical trials

Osilodrostat is an oral inhibitor of 11 beta-hydroxylase (CYP11B1) that blocks the hydroxylation of deoxycortisol to cortisol, the final step in cortisol synthesis (Fig. 1). Osilodrostat, first studied in patients with hyperaldosteronism, also inhibits the activity of aldosterone synthase (CYP11B2), decreasing aldosterone synthesis [102]. In these patients, a reduction in circulating cortisol levels and adrenal insufficiency was observed, in addition to the expected fall in aldosterone levels.

Osilodrostat shares a similar mechanism of action with metyrapone. However, it is significantly more potent, having a 3-fold higher affinity to 11 beta-hydroxylase than metyrapone, as demonstrated in preclinical studies (in vitro half maximal inhibitory concentration (IC_50_) of 7.5 nM vs 2.5 nM for metyrapone) [103]. In addition, its longer half-life allows twice daily oral administration, conferring an advantage over metyrapone, which may require administration up to 6 times daily [103, 104]. In a 10-week, proof of concept study (LINC-1; NCT01331239), osilodrostat demonstrated good efficacy, normalizing UFC in 11 of 12 patients with CD. Mean ACTH, 11-deoxycortisol and 11-deoxycorticosterone levels increased during treatment but declined after discontinuation [103]. The extension phase study enrolled 4/12 of the original cohort and an additional 15 new patients with CD with UFC more than 1.5 × upper limit of normal (ULN). Patients received 4–10 mg per day as a starting dose depending on the degree of UFC elevation. Doses were then adjusted every 2 weeks as needed to 10–60 mg/day until UFC normalized. UFC normalized in 78.9 % of patients at 22 weeks [105]. Adverse reactions were well tolerated, the most common of which were nausea, diarrhea and asthenia. Adrenal insufficiency and/or glucocorticoid withdrawal was reported in one-third of patients, highlighting the potency of osilodrostat and the need for slow and careful dose titration. Elevated testosterone levels, hirsutism and acne were observed in 3 of 11 females (Table 1). Importantly, no new safety signals were identified after 22 weeks of treatment. Interim analysis of the open-ended extension study (LINC-2) points to sustained long-term efficacy of osilodrostat. At 19 months, mean UFC and 0800 morning serum cortisol levels were within the normal range, an effect that had been observed since week 6. Overall response rate was 93.8 % at week 22 and at month 19, with no treatment escape noted in any patients. Improvement was noted in weight and body mass index (BMI) [106]. Based on these results, osilodrostat shows promise as an alternative treatment in CD [104] and two phase III studies (LINC-3 and LINC-4) are currently underway.

Recently, the combination of osilodrostat and pasireotide was evaluated in rats [107]. Acceptable safety and efficacy was shown and lend interest to future testing of this combination in clinical trials.

### Levoketoconazole (COR-003)

Levoketoconazole is an investigational drug for CS that acts similarly to its enantiomer, ketoconazole (2S,4R and 2R,4S racemic mixture), but is hypothesized to provide better efficacy and safety. The (2S,4R) single enantiomer, levoketoconazole is 2 and 7 times more potent than ketoconazole in inhibiting 21-hydroxylase (CYP21) and 17 alpha-hydroxylase (CYP17), and 11 beta-hydroxylase (CYP11B1) steroidogenesis enzymes, respectively (Fig. 1). A recent pre-clinical study also confirmed a significantly higher potency in enzyme inhibition than its pure (2R, 4S) enantiomer. This theoretically leads to lower doses, and fewer adverse events [104].

Experiments in an animal model showed greater potency and efficacy in reducing serum corticosterone. In a phase I study, when administered to healthy subjects, levoketoconazole (400 mg/day) reduced serum cortisol levels significantly by day 4, compared to placebo and ketoconazole. The drug was well tolerated; headache, back pain and nausea were the most frequently reported adverse events [108]. Being 12 times less potent than ketoconazole in inhibiting CYP7A1, less interference with bile acid synthesis and metabolite elimination, and therefore less hepatoxicity, is anticipated [108]. Reduction of cholesterol and C- reactive protein have also been reported in patients with type 2 diabetes mellitus after short-term administration of levoketoconazole [109]. A phase III single-arm, open-label trial (clinicaltrials.gov; NCT01838551) is currently underway to evaluate the efficacy, safety, tolerability and pharmacokinetics of levoketoconazole in patients with CS [110].

### R-Roscovitine

The cyclin-dependent kinase (CDK) and cyclin E inhibitor R-roscovitine has been evaluated as potential therapy for CD patients. R-roscovitine was first shown to be effective in reducing ACTH and serum corticosterone levels in a mouse model. Further studies demonstrated restraint of tumor growth after R-roscovitine treatment in a mouse model with xenografted ACTH-secreting pituitary tumor [111]. R-roscovitine induces corticotroph tumor cell cycle arrest. Its mechanism of action is still under study, and seems to be related to disruption of cyclin E2F1 binding to proopiomelanocortin (POMC) gene promoter and suppression of corticotroph transcription factors, resulting in decreased ACTH expression (Fig. 1) [112]. Currently, a phase II clinical trial (clinicaltrials.gov; NCT02160730) in human patients with CD is ongoing to evaluate the efficacy and safety of R-roscovitine (Table 1).

### Retinoic acid

Retinoic acid is a nuclear receptor ligand that was proposed as a treatment for CD after it was shown to decrease ACTH secretion and corticotroph tumor growth in vitro and in animal models, via inhibition of POMC expression in corticotroph tumors (Fig. 1) [113]. The first proof-of concept clinical study demonstrated ≥ 50 % reduction in UFC in 5 of 7 patients after 6 months of treatment with 10–80 mg/day of retinoic acid; UFC normalized in three patients [114]. A recent open-label clinical trial demonstrated that 20–80 mg/day of the 13-cis isomer of retinoic acid, isotretinoin, resulted in UFC normalization in 4 of 16 patients (25 %) at 12 months, with UFC reductions up to 52 % seen in the rest [115]. Adverse events, though mild and reversible, were encountered in more than 40 % of patients. Further randomized, double-blind, clinical trials are needed to evaluate its efficacy in patients with CD.

## Medical treatments on the horizon

### Epidermal growth factor receptor (EGFR) inhibitors

EGFR is expressed in the pituitary, but mainly in corticotroph adenomas, and plays an important role in tumorigenesis. Inhibition of ACTH secretion, reduction in tumor size and clinical improvement were observed in animal models treated with gefitinib, an EGFR tyrosine kinase inhibitor [116]. Recently, an additional mechanism by which EGFR plays a role in the pathophysiology of CD has been proposed, with potential therapeutic applications [117]. The ubiquitin-specific peptidase eight (USP8) gene was found to be mutated in 33–66 % of patients with CD [118]. Mutations clustered around the 14-3-3 protein that enhances the catalytic activity of USP8. As a result of these mutations, EGFR deubiquitination occurs, impairing its downregulation and increasing its activity, signaling and ACTH secretion. Such mutations have also been found to increase SSTR5 expression, and patients with these mutations may therefore, show a better response to pasireotide. This has potential therapeutic implications and warrants further investigation.

### Chimeric compounds

By working synergistically, chimeric compounds that interact with both dopamine D2R and SSTRs have the potential to be more potent than individual compounds in controlling tumor growth and ACTH release [119, 120]. The chimeric compound BIM-23A760, which has high affinity for SSTR2, D2R and to a lesser extent SSTR5, was however, found to produce interfering metabolites that compete with the activity of the drug itself, decreasing its efficacy. Further development of second-generation chimeric compounds is currently under way [121]. BIM-065, which has potent bioactivity at both the SSTR2 and D2R, has recently been developed. While it does have high binding affinity to SSTR5, it has a lower bioactivity, than pasireotide. Interestingly however, this compound was shown to increase insulin sensitivity in normal rats [122].

### Selective, peptide melanocortin-2 receptor antagonists

Recently, several peptides that act as ACTH antagonists at the melanocortin-2 receptor (MC2R) have been discovered. One particular compound, IRC-274, has been observed in in vitro studies to inhibit ACTH production in human embryonic kidney (HEK) 293 cells co-expressing both MC2 and the melanocortin-2 receptor accessory protein (MRAP), and in two different rodent models, to reduce circulating corticosterone levels in a dose and time-related manner [123, 124]. These observations could lead to the development of a novel therapeutic agent for the normalization of cortisol levels in CD patients (Fig. 1).

## Conclusions

Medical treatment for CD is an expanding and dynamic field. Recent studies describing novel treatment strategies have shown promising results, expanding the role for medical therapy in the treatment of CD. New insights into the pathophysiology of CD have led to new targets for drug development aimed at decreasing ACTH secretion. Currently available adrenal steroidogenesis inhibitors are useful especially in the short-term control of cortisol excess but are associated with treatment escape, multiple adverse effects and drug interactions. Pituitary-directed drugs acting at the level of the corticotroph adenoma have the potential advantage of tumor shrinkage. While data for the use of cabergoline is limited, pasireotide and mifepristone have demonstrated good efficacy and tolerability. Combination therapy is an additional strategy that aims to increase treatment efficacy, whilst minimizing adverse events. In particular, the combination of pasireotide, cabergoline and ketoconazole has shown promise and larger studies are awaited. Novel therapies such as osilodrostat and levoketoconazole offer hope of increased efficacy and R-roscovitine, retinoic acid, EGFR inhibitors, second-generation chimeric compounds and selective, peptide MC2R antagonists are emerging therapies currently under evaluation. Ultimately, individualized treatment is needed, depending on the severity of hypercortisolemia, tumor size and comorbidities.

## Abbreviations

ACTH: Adrenocorticotropin hormone
CD: Cushing’s disease
CS: Cushing’s syndrome
CYP3A: Cytochrome P450 3A
D2R: Dopamine type 2 receptor
DPP-4: Dipeptidyl peptidase 4
EGFR: Epidermal growth factor receptor
GABA: Gamma-amino butyric acid
GLP-1: Glucagon-like peptide-1
GR: Glucocorticoid receptor
LAR: Long-acting repeatable
MC2R: Melanocortin type 2 receptor
MRI: Magnetic resonance imaging
POMC: Proopiomelanocortin
PPAR-gamma: Peroxisome proliferator-activated receptor gamma
sc: Subcutaneous
SEISMIC: Study of the efficacy and safety of mifepristone in the treatment of endogenous Cushing’s syndrome
SRL: Somatostatin receptor ligand
SSTR: Somatostatin receptor
USP8: Ubiquitin specific peptidase eight
